# Dynamic interaction between basin redox and the biogeochemical nitrogen cycle in an unconventional Proterozoic petroleum system

**DOI:** 10.1038/s41598-019-40783-4

**Published:** 2019-03-26

**Authors:** Grant M. Cox, Pierre Sansjofre, Morgan L. Blades, Juraj Farkas, Alan S. Collins

**Affiliations:** 1 University of Adelaide, Adelaide, S.A. 5005 Australia; 2 UMR 6538, Laboratoire Géosciences Océan, Institut Universitaire Européen de la Mer, rue Dumont d’Urville, 29280 Plouzané, France

## Abstract

Precambrian hydrocarbons and their corresponding source rocks are distinctly different from their Phanerozoic counterparts, having been deposited in persistently anoxic environments in ecosystems dominated by bacteria. Here, we show that cyclic enrichment of organic matter in the world’s oldest hydrocarbon play (ca. 1.38 Ga), is not associated with flooding surfaces and is unrelated to variations in mineralogy or changes in the relative rate of clastic to biogenic sedimentation—factors typically attributed to organic enrichment in Phanerozoic shales. Instead, the cyclic covariation of total organic carbon, *δ*^15^N, *δ*^13^C and molybdenum are explained by the feedback between high levels of primary productivity, basin redox and the biogeochemical nitrogen cycle. These factors are important in constraining productivity in the marine biosphere, the development of Precambrian hydrocarbon source rocks, and more generally in understanding oxygenation of the ocean and atmosphere through Earth history; as all are ultimately related to organic carbon burial.

## Introduction

High total organic carbon (TOC) content within sediments has been attributed to various factors, including high primary productivity^1^, high nutrient fluxes due to warm and wet climatic conditions^2,3^, basin redox conditions facilitating the preservation of organic matter^4^, mineralogical controls on organic carbon export^5–7^ and changes in the relative rate of clastic to biogenic sedimentation^8^. Understanding these processes, which likely vary in both space and time, are important in constraining the development of hydrocarbon source rocks and more generally in understanding oxygenation of the ocean and atmosphere through Earth history; as both are tied to organic carbon burial.

Primary productivity is generally accepted to be limited by the availability of phosphorus, dissolved inorganic nitrogen (DIN: NH_4_^+^, NO_3_^−^) and bio-essential trace metals^9–12^. Furthermore, it is widely appreciated that high levels of primary productivity and organic matter export can influence basin redox. In particular, Johnston *et al*.^13^ demonstrated the relationship between high levels of organic carbon export and euxinia, while Anbar and Knoll^11^ highlighted how basin redox, in particular euxinia, influences nutrient concentrations, in particular trace nutrients such as molybdenum, with flow on effects for bioavailable nitrogen. Furthermore, recent work has shown that the magnitude of organic matter supply also affects the rate of denitrification^14^ and consequently bioavailable nitrogen concentrations. Such effects on nitrogen availability naturally affect primary productivity and organic carbon burial, as biologically available nitrogen can limit photosynthesis and is often referred to as the proximal limiting nutrient^10^. Building upon these general observations, we present 𝛿^15^N and 𝛿^13^C data from the hydrocarbon-bearing Roper Group of the greater McArthur Basin of northern Australia. These suggest that the cyclic production of high TOC sediments in this Mesoproterozoic black shale succession is controlled by oscillations between nitrogen replete and nitrogen limited conditions. These are hypothesized to be a consequence of the coupling of elevated primary productivity, high degrees of organic carbon export, basin redox and the biogeochemical nitrogen cycle. These processes interact to impact nitrogen availability through changes in the balance between nitrogen supply via N_2_ fixation and nitrogen loss through denitrification.

## Nitrogen Isotopes

In models for stratified oceans with only a shallow oxic layer^15,16^, a condition widely considered to characterise the Proterozoic^17^, the availability of bioavailable nitrogen would be tied to prokaryotes (i.e. cyanobacteria) that perform N_2_-fixation (i.e. diazotrophy), along with remineralised nitrogen. The loss of bioavailable nitrogen is achieved either through heterotrophic denitrification and/or anaerobic ammonium oxidation (i.e. ANAMMOX). It is widely held that diazotrophs are at a competitive disadvantage under nitrogen replete conditions. Consequently, it is only under nitrogen-limited conditions that diazotrophs are expected to fix appreciable amounts of nitrogen. As the diazotroph biomass has an average 𝛿^15^N of ~−1‰, and can range from −3‰ to +1‰^18^, any increase in N_2_-fixation in response to nitrogen limitation tends to decrease the 𝛿^15^N of DIN toward pure N_2_-fixation values (i.e. −3‰ to +1‰). In contrast to N_2_ fixation, denitrification and anaerobic ammonium oxidation preferentially return light nitrogen to the atmosphere, leaving the residual pool of bioavailable nitrogen isotopically heavier^19,20^. Nitrification and biological assimilation produce large fractionations in nitrogen isotopes^21,22^, however, these fractionations are ordinarily not expressed, as such processes are nearly quantitative. This is observed in the modern marine system through the 1:1 correspondence between 𝛿^15^N_sedimentary_ and 𝛿^15^N_nitrate_ (e.g. Fig. 34.6 – ref.^23^). Therefore, nitrogen isotopes typically trace the balance between N_2_-fixation and heterotrophic denitrification and anaerobic ammonium oxidation, with the latter two being highly redox dependent being carried out under sub-oxic conditions^24–26^.

## Geology

**Figure 1:**
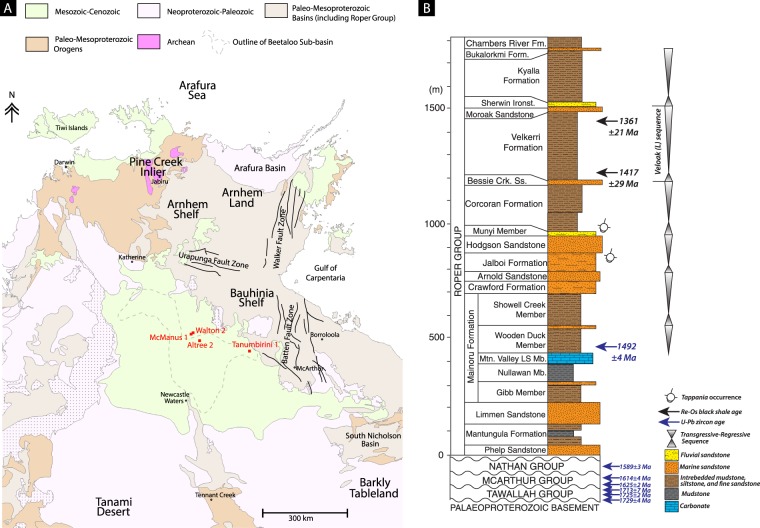
Regional geology and stratigraphic sequence. (**A**) Distribution of sedimentary basins of northern Australia. (**B**) Stratigraphic column for the Roper Group showing major transgressive-regressive sequences. Reproduced from ref.^36^.

The Roper Group of northern Australia, is the younger of four unconformity-bound sedimentary packages of the much larger greater McArthur Basin^27,28^. Significant lateral thickness changes occur within the Roper Group. It is thin (~1–2 km) in the vicinity of the east-west trending Urapunga Fault Zone, of moderate thickness over the Broadmere Inversion Structure (~2 km^29^), and thinnest (<500 m) over the north–south trending Batten Fault Zone (Fig. 1A)^28,30–32^. Southwest of the Batten Fault Zone, the Roper Group thickens to >5 km in the Beetaloo Sub-basin^28,30–33^ (Fig. 1A), which is interpreted to represent the main depo-centre of the Roper Seaway^30,32^.

The Velkerri Formation, a black-shale dominated unit within the Roper Group, is a key environmental archive for the early–middle Mesoproterozoic due to its exceptional thickness, well-constrained age (1386 ± 13 Ma to 1361 ± 21 Ma^34,35^), large variations in organic carbon content, and low metamorphic grade^36,37^. Prior work has shown that the Velkerri Formation hosts some of the oldest known ‘live‘ hydrocarbon occurrences^38^, and continues to generate substantial interest as an unconventional gas play, with an estimated in place gas resource of 118 trillion cubic feet^37^. Recent production testing has resulted in the successful hydraulic fracturing of this Mesoproterozoic formation resulting in substantial gas flows^39^ making this one of the oldest proven unconventional hydrocarbon plays.

The Velkerri Formation is the initial deep water facies of the Veloak sequence^30^ (Fig. 1B), which comprises the dominantly basinal facies of the Velkerri Formation transitioning up-section into cross-bedded sandstones of the Moroak Sandstone (Fig. 1B). The Velkerri Formation is formally divided into three distinct members, the Kalala (lower), Amungee (middle) and Wyworrie (upper) members^40^. The Amungee Member is the principal black shale facies with total organic carbon contents reaching ~10%^36,37^.

**Figure 2:**
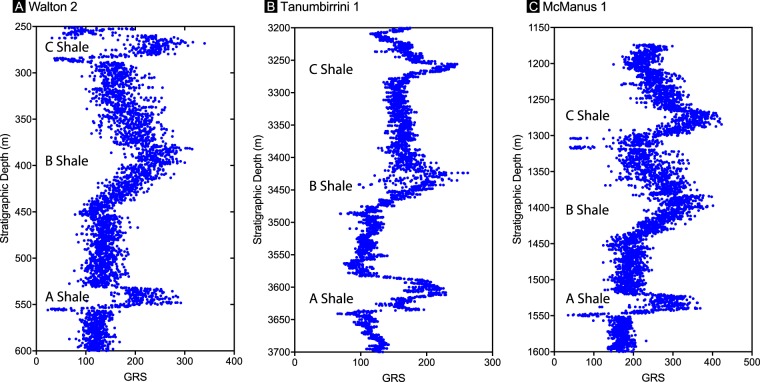
Gamma ray spectra (GRS) for regionally distributed sections of the Velkerri Formation. See Fig. 1 for well locations.

**Figure 3:**
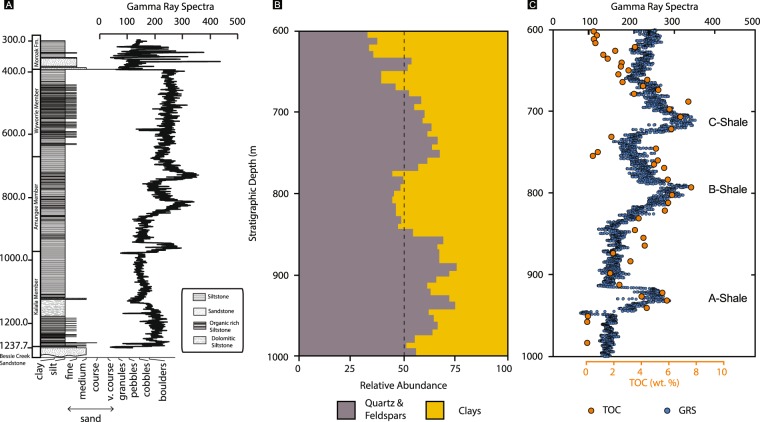
(**A**) Stratigraphic log of the Altree 2 well along with full gamma ray spectra. (**B**) Relative abundance of major mineral groupings based on high resolution XRD analysis of the Altree 2 well. (**C**) TOC overlain on expanded gamma ray spectra for Altree 2.

Detailed sedimentary petrology of the Amungee Member has shown that the facies was deposited below storm weather wave base and is composed of thinly laminated, grey-green to dark grey clays, pale grey silts and rare, fine-grained sands^40,41^. Organic enrichment within the Amungee Member occurs principally as three prominent horizons that are informally referred to as the A, B and C organofacies. Gamma Ray Spectra (GRS) reveals that these organofacies are a feature of the Amungee Member at the regional scale (Fig. 2). The organic-rich and organic-poor intervals show no systematic relationship to either grain size or minerology (Fig. 3). Based on this observation it was noted that the organic-rich and organic-poor intervals do not reflect changes in the energy of deposition or water depth, but must reflect changing water column biochemistry^41^.

## Methods

### Samples

Samples were obtained from the petroleum well Altree-2 drilled by Pacific Oil and Gas and now housed in the Northern Territory Geological Surveys core facility in Darwin, N.T. The Velkerri Formation, which forms the basis of this study was intercepted at depths between 391.72 m and 1229.65 m. Rock samples were broken into ~2 cm^3^ fragments and the resulting chips where milled in a tungsten carbide mill until the powder could pass through a 75 µm mesh resulting in a homogenous sample powder.

### Bulk rock pyrolysis

Pyrolysis measurements were undertaken using a Weatherfords SRA. Crucibles were loaded into the carousel and heated under inert nitrogen in both the pyrolysis (to obtain S1, S2, T_max_ and S3 peaks) and oxidation ovens (to obtain the S4 peak). The pyrolysis oven was first held at 300 °C for 5 minutes and ramped at 25 °C per minute from 300 °C to 650 °C. The oxidation oven was held at 400 °C for 3 minutes, ramped at 20 °C per minute from 400 °C to 850 °C and held at 850 °C for 5 minutes. The flame ionisation detector (FID) was calibrated by running IFP standard ‘160000’ in ‘standard’ mode. The IR Analysers were calibrated against standard gas with known concentration of CO_2_ and CO. An analysis blank was run as ‘blank’ mode with the sample batch and the blank data was automatically subtracted from all analyses. An internal standard was also run first with each batch to ensure the instrument status. The results were processed with Optkin 3.0 software where peak heights and geochemical indices including Total organic carbon (TOC), Oxygen Index (OI), Hydrogen Index (HI) and Production Index (PI) are automatically calculated.

### XRD analysis

X-ray diffraction (XRD) analysis was undertaken on the raw powder utilising a Bruker D4 XRD. Quantification of the multiphase mixtures using Rietveld quantitative analysis was undertaken using the DIFFRAC™ software suite.

### Carbon and nitrogen stable isotope analyses

1 to 2 grams of sample powder were treated with excess 3N HCl for 24 hours to remove trace carbonate minerals. Insoluble residual was centrifuged and rinsed with deionized water until supernatant achieved a pH of 4. Isotopic analyses for nitrogen and carbon were performed using a Euro EA Elemental Analyzer coupled to a Nu Horizons continuous-flow stable isotope mass spectrometer. Analyses were performed in the stable isotope laboratory at the University of Adelaide. Powdered, decarbonated samples were weighed and sealed in tin capsules for isotopic analysis and were combusted at 1050 °C. Data are reported using delta notation relative to atmospheric N_2_ for nitrogen and the Vienna Pee Dee Belemnite International Standard (V-PDB) for carbon. 𝛿^15^N were calibrated to international reference standards IAEA N1, N2, NO-3, and USGS 25, 32, 40 and 41. 𝛿^13^C was calibrated to IAEA CH-6 and CH-7; USGS 24, 40, and 41; and NBS-22. Standard deviation for bulk samples is 0.15 for 𝛿^13^C and 0.1 for 𝛿^15^N.

## Results

**Figure 4:**
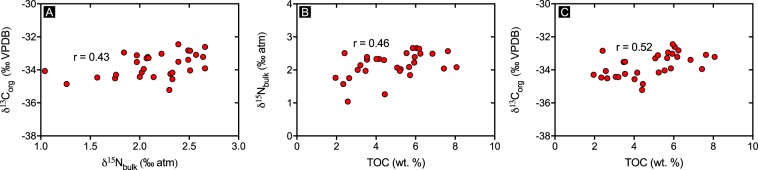
(**A**) 𝛿^13^C_org_ vs. 𝛿^15^N_bulk_, (**B**) 𝛿^15^N_bulk_ vs. TOC and (**C**) 𝛿^13^C_org_ vs. TOC. R values are Spearman Correlation co-efficient and all are significant at α = 0.05. Two tailed p values are 0.0182, 0.0112 and 0.0036 respectively.

**Figure 5:**
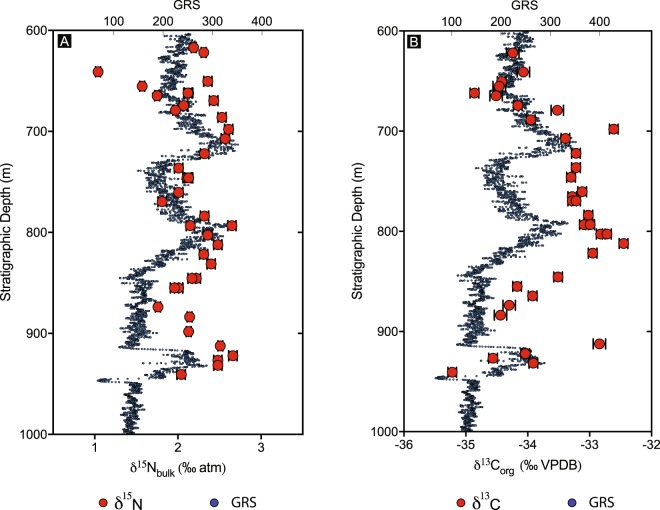
Up-section variations in 𝛿^15^N_bulk_ (**A**) and 𝛿^13^C_org_ (**B**) along with a gamma ray spectrum.

We analysed 30 samples (Figs. 4 and 5) from the Velkerri Formation at a sampling resolution of 1 sample every ~11m. 𝛿^15^N_bulk_ varies from +1.04‰ to +2.67‰ while 𝛿^13^C_org_ varies from −35.2‰ to −32.4‰. Statistically significant correlations exist between 𝛿^15^N_bulk_, 𝛿^13^C_org_, and TOC (at α = 0.05). Atomic C:N ratios range from 17.2 to 49.8.

### Alteration

**Figure 6:**
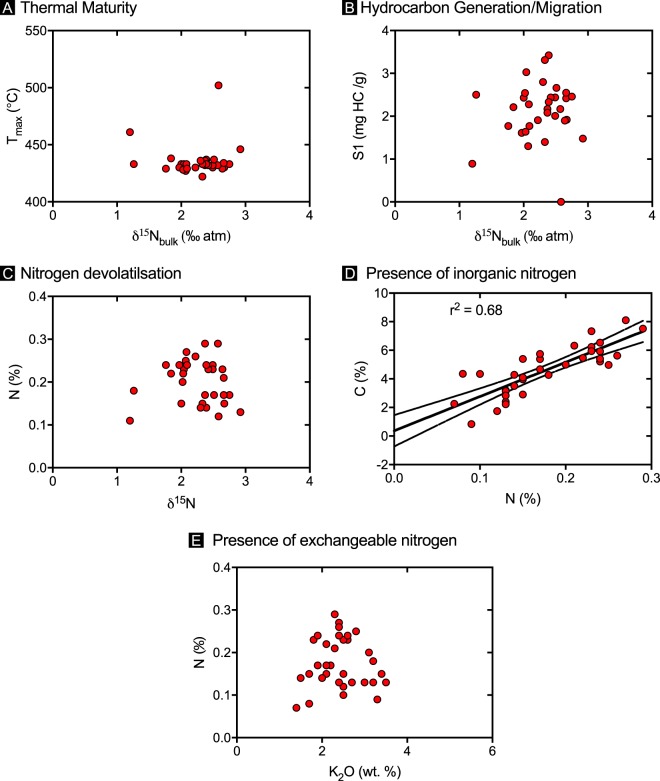
Possible secondary alteration of primary 𝛿^15^N signatures due to (**A**) thermal maturity, (**B**) hydrocarbon generation and/or migration, (**C**) nitrogen devolatilisation, (**D**) presence of inorganic nitrogen and (**E**) exchangeable nitrogen.

While it has been shown that changes in the nitrogen isotope characteristics of organic matter can occur during particle sinking and diagenesis (e.g. ref.^42^), it has also been shown that in high sediment accumulation zones, such as continental margins, no significant alteration of primary 𝛿^15^N occurs^43–45^. Furthermore, unaltered bulk sedimentary 𝛿^15^N generally reflects the 𝛿^15^N of bioavailable nitrogen^23^. While such work provides confidence in the fidelity of 𝛿^15^N of organic-rich sediments, we have investigated the possibility of alteration, focussing on relationships between 𝛿^15^N and thermal maturity, hydrocarbon generation, migration and devolatilisation (Fig. 6A–C), and find no evidence for the systematic alteration of 𝛿^15^N via these processes. Furthermore, relationships between nitrogen abundance (N%), carbon abundance (C%) (Fig. 6D) and K_2_O concentrations (%) (Fig. 6E) suggest that sources of inorganic nitrogen or exchangeable NH_4_^+^ are negligible.

## Discussion

### Basin dynamics, primary productivity, redox, trace nutrients and the biogeochemical nitrogen cycle

**Figure 7:**
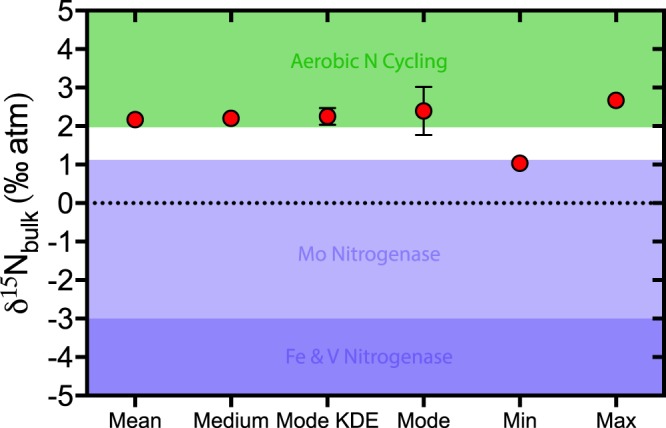
Measures of central tendency with 95% confidence intervals along with absolute minimum and maximum 𝛿^15^N values. Field for Mo-Nitrogenese biomass is from ref.^18^.

Measured nitrogen isotopes for the Velkerri Formation vary between +1.04‰ and +2.67‰, while measures of central tendency for this basinal facies have values that fall close to +2‰ (Fig. 7). Only a single sample has an isotopic value of ~+1‰, a value that reasonably could be attributed solely to N_2_ fixation (i.e. −3‰ to +1‰^18^), all other samples have 𝛿^15^N values ≥+2‰, which are values consistent with aerobic nitrogen cycling^16^. Significantly, these variations in 𝛿^15^N are not random but show statistically significant cycling at the 95% confidence level at a wavelength of ~110m (see Supplementary Information) and show consistent positive covariation with 𝛿^13^C_org_, TOC (Fig. 4) and GRS (Fig. 5). Considering that these cyclic changes in TOC and GRS are a regional feature of the basin, variations in 𝛿^15^N and 𝛿^13^C_org_ provide a basis for understanding the development of the observed cyclic organofacies.

#### Lateral gradients in bioavailable nitrogen

**Figure 8:**
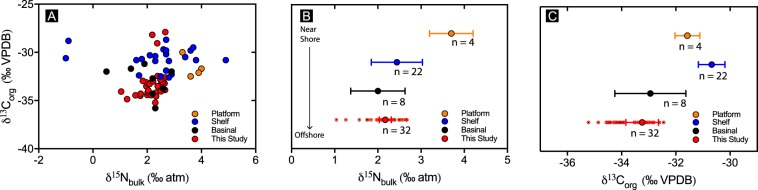
(**A**) 𝛿^13^C vs. 𝛿^15^N. (**B**,**C**) Average and 95% confidence intervals for platform (yellow), shelf (blue), and basinal (black) facies for the Roper Group (ref.^47^). Red data are from this study.

One possible explanation for the up-section variation we observe in 𝛿^15^N could be that they represent lateral gradients in 𝛿^15^N from near shore to offshore environments. Stüeken^46^ argued for a lateral gradient in 𝛿^15^N based on samples from the Mesoproterozoic Belt Supergroup. In this study, 𝛿^15^N values varied from ~−1‰ in offshore basinal facies to ~+5‰ in near-shore facies. This trend was interpreted to reflect a transition from more productive coastal environments with active aerobic nitrogen cycling to nitrate poor offshore environments dominated by nitrogen fixation. A similar study for the Roper Group^47^ identified a similar lateral gradient between platform (n = 4) and basinal (n = 8) facies, however, unlike the Belt Basin, 𝛿^15^N values between basinal (n = 8) and shelf (n = 22) facies were statistically indistinguishable (Fig. 8).

Due to the near-shore–offshore lateral gradients observed in 𝛿^15^N in some Mesoproterozoic basins^46,47^, it raises the possibility that the cycles observed in 𝛿^15^N (Fig. 5A) represent subtle changes in water depth reflecting subtle changes in a lateral 𝛿^15^N gradient. However, we think this is unlikely, as neither this study or previous studies^30,41^ have shown any evidence for changes in water depth within the Amungee Member of the Velkerri Formation. Furthermore, the overall coarsening-upward sequence of the Velkerri Formation is well documented^30,36,41^ and a prediction of this near shore-offshore gradient would be that both 𝛿^15^N and 𝛿^13^C should be isotopically heavier up-section (e.g. Fig. 8B & C), this is not observed (Fig. 5). However, our basinal facies are isotopically identical to basinal Velkerri samples from the wider basin and distinct from shallow water facies, this would indicate that the organic enrichment of the Amungee Member of the Velkerri Formation is unlikely to be due to organic matter transported from shallower (i.e. shelf or platform, Fig. 8) near-shore environments, contrasting with previous suggestions^33^.

#### Partial assimilation and/or partial nitrification

If lateral variations do not provide a satisfactory explanation for the observed 𝛿^15^N values, what mechanism can produce the observed range in 𝛿^15^N, especially values greater than +2‰? Partial assimilation of bioavailable nitrogen could generate isotopically heavy 𝛿^15^N as partial assimilation of NH_4_^+^ leaves behind a residual pool of isotopically heavy nitrogen^48^. Furthermore, as assimilation of bioavailable nitrogen goes to completion in the modern nitrogen limited ocean^42,49^, it is unclear why partial assimilation would be a widespread characteristic considering the low levels of dissolved molybdenum estimated for the Mesoproterozoic^50^. Furthermore, modern analogues of Mesoproterozoic basins, such as the Black Sea and Cariaco Basins, also do not preserve evidence for partial assimilation^49,51^.

Partial nitrification of remineralised NH_4_^+^ also has the ability to generate a ^15^N enriched residual pool^52^. However, considering that nitrification is rapid and occurs at vanishingly low levels of O_2_^53^, it is expected to go to completion as nitrogen is widely considered the proximal limiting nutrient^10,12^.

#### Denitrification

A possible candidate to produce isotopically heavy 𝛿^15^N would be partial denitrification either via heterotrophic denitrification and/or anaerobic ammonium oxidation. As denitrification occurs under low oxygen conditions, and water column redox is, in part, determined by the export and remineralisation of organic carbon from overlying surface waters, a dynamic feedback between organic matter production, export, remineralisation and denitrification can be envisaged. Furthermore, recent experimental results have shown that the rate of denitrification increases with increasing organic matter flux through the supply of fresh labile organic matter^14^. Such a dynamic feedback would provide a viable mechanism for producing cyclic enrichments in organic carbon, similar to those observed in the Velkerri Formation.

#### Reduction in nitrogen fixation

A further mechanism for generating isotopically heavy nitrogen was proposed by Anbar and Knoll^11^, who speculated that the redox sensitive behaviour of molybdenum, coupled with its role as a cofactor associated within MoFe-nitrogenase, the dominant and most efficient enzyme responsible for nitrogen fixation in diazotrophs^54–56^, may have implications bioavailable nitrogen (Mo-N co-limitation).

The basis for Mo-N co-limitation is that under oxic conditions molybdenum occurs as the soluble molybdate ion (MoO_4_^2−^), however, under euxinic conditions, molybdenum reacts strongly with hydrogen sulphide to form the particle reactive thiomolybdate ion^57^. Thiomolybdates can be removed from seawater in organo-metallic complexes or within sulphide phases, resulting in molybdenum limitation. Recent work has shown that N_2_ fixation rates are reduced by ~80% at dissolved molybdenum concentrations between 1–5 nM^58,59^, such values are in the predicted range of dissolved molybdenum concentrations of Mesoproterozoic oceans^50^.

While it has been argued that euxinia and nitrate are mutually exclusive^60^, Stüeken *et al*.^16^ pointed out that isotopically heavy 𝛿^15^N has been documented in numerous Archean and Proterozoic basins inferred to have experienced euxinia, and suggested nitrate and euxinia are likely compatible in highly stratified waters. Euxinic conditions have been suggested in the Roper basin^36,61^. In particular, Cox *et al*.^36^ noted enrichment in molybdenum in Velkerri sediments (up to ~100 ppm, twice the Mesoproterozoic average^50^) while biomarker studies^62^ identified dibenzothiophene within the Amungee Member which at the very least suggests sulfate reduction within the sediments, however, together support transient deep-water euxinic conditions^57,63–65^.

**Figure 9:**
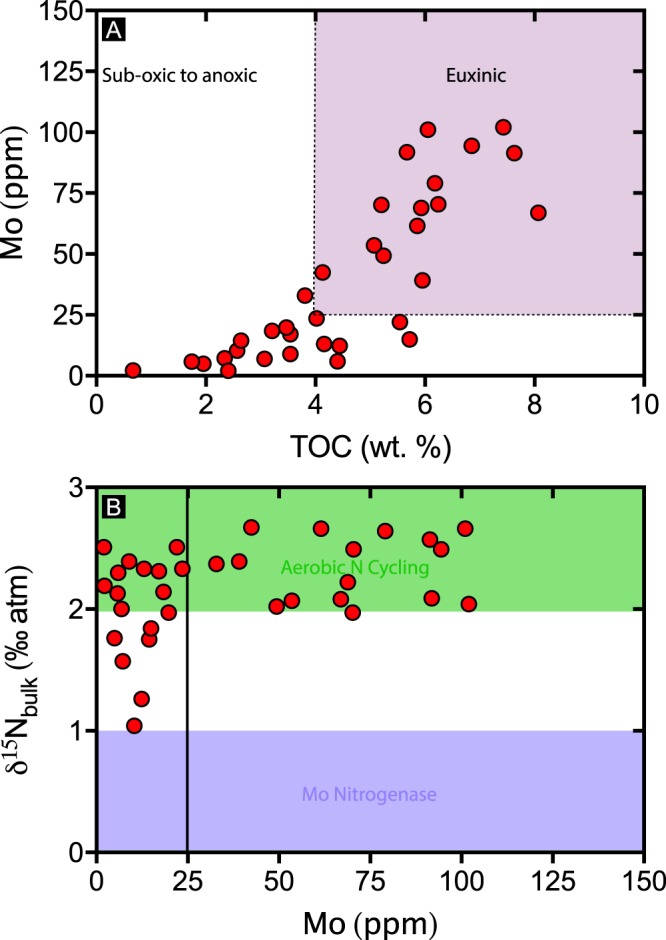
(**A**) Molybdenum vs. TOC and (**B**) 𝛿^15^N vs. Mo. Euxinic conditions are interpreted to exist at molybdenum concentrations above 25 ppm and TOC above 4% from ref.^36^.

Based on the boundaries for euxinia defined for the Velkerri Formation (~25 ppm Mo and ~4% TOC^36^), the comparison between molybdenum and 𝛿^15^N reveals two distinct populations (Fig. 9). Samples with molybdenum concentrations below 25 ppm show variations in 𝛿^15^N between ~+1‰ to ~+2.5‰. In contrast, samples containing more than 25 ppm molybdenum have 𝛿^15^N exclusively above ~+2‰. Both a 2-tail *t*-test and Wilcoxon Signed Rank Test reveal that these two populations are statistically distinct (p = 0.0002 at α = 0.05).

Typically, the shifting balance between nitrogen supply via N_2_ fixation (source of isotopically light 𝛿^15^N) and its loss via denitrification (producing isotopically heavy 𝛿^15^N residual pool) is thought to be largely governed by basin redox conditions^15,16,66^. The dichotomy in the distribution of 𝛿^15^N and molybdenum (Fig. 9) suggests that in those samples containing >25 ppm molybdenum, the balance between N_2_ fixation and denitrification was heavily biased towards denitrification. Considering the evidence for euxinia^36,62^, a parsimonious explanation would be molybdenum limitation affecting N_2_ fixation rates.

#### Dynamic interplay between primary producers, basin redox and nitrogen cycling

Partial assimilation and/or nitrification seem unlikely candidates controlling the variations observed in our 𝛿^15^N record. Furthermore, while the range of 𝛿^15^N values are completely consistent with values associated with offshore basinal facies (e.g. refs.^46,47^), the cyclic variations cannot be explained by this mechanism, as it is incompatible with the detailed sedimentology of the Amungee Member and the overall high stand systems track for the Velkerri Formation.

The model proposed here envisages that during periods of high nutrient supply, as argued for the Amungee Member^36^, a cyclic process developed with high primary productivity and burial driving increasing levels of denitrification (i.e. ref.^14^). This process is suggested to have led to overall nitrogen limitation as denitrification ultimately depletes bioavailable nitrogen. However, the dichotomy in the distribution of 𝛿^15^N and Mo (Fig. 9) and evidence for deep water euxinia suggests that a reduction in the concentration of dissolved molybdenum likely restricted N_2_ fixation as well through Mo-N co-limitation.

The consequence of increased denitrification and reduced N_2_ fixation is a reduction in primary productivity due to nitrogen limitation, a reduction in organic matter export/remineralisation and an easing of euxinic conditions. This ultimately resulted in increased levels of dissolved molybdenum, which shifted the balance towards renewed N_2_ fixation. Such a sequence of events is interpreted to have produced the cyclic variations between light and heavy 𝛿^15^N along with positive covariations in TOC and 𝛿^13^C seen in the Amungee Member.

While this discussion has centred around nitrogen, carbon isotopes also support this model. For the same reasons given for variations in 𝛿^15^N, 𝛿^13^C variations do not reflect lateral environmental gradients in 𝛿^13^C. They exhibit a positive correlation with TOC and as peaks in 𝛿^15^N and TOC are associated with the most isotopically heavy 𝛿^13^C, it is likely that these 𝛿^13^C variations reflect changes in dissolved inorganic carbon, or more specifically, variations in contemporaneous organic carbon burial (ƒ_org_), which likely reflect the strength of primary productivity. These systematic positive covariations (𝛿^15^N, 𝛿^13^C, TOC, Mo) can be explained, ultimately, by changes in the balance between N_2_ fixation and denitrification resulting from the dynamic interaction between primary productivity, basin redox and biological metabolisms associated with nitrogen cycling. This biogeochemical model does not preclude higher order controls on the observed cyclicity such as orbital forcing mechanisms, however, available age constraints do not allow for a robust testing of such a higher order mechanism.

## Conclusions

Cyclic behaviour in organic matter enrichment within the Mesoproterozoic Velkerri Formation is not associated with maximum flooding surfaces or to variations in mineralogy or clastic flux. Nitrogen and carbon isotopes along with molybdenum concentrations record the shifting balance between N_2_ fixation and denitrification pathways which can be related to high levels of organic carbon production, export and variations in basin redox, specifically shifts between sub-oxic to euxinic conditions. The requirement of molybdenum in biological N_2_ fixation along with the efficient scavenging of molybdenum under euxinic conditions results in Mo-N co-limitation and a decrease in primary productivity. These feedbacks highlight the dynamic interplay between primary producers, basin redox and biogeochemical nitrogen cycling. Such interactions are important in constraining productivity in the marine biosphere and highlight that high levels of primary productivity and euxinia are inherently self-limiting. Furthermore, such feedbacks are important in understanding the development of Precambrian hydrocarbon source rocks, and more generally in understanding oxygenation of the ocean and atmosphere through Earth history; as all are ultimately related to organic carbon burial.

## Supplementary information

Supplementary Information

## Data Availability

All data that underlies this study can be found in Tables 1 through 3 in the accompanying supplementary information. Details of the well Altree-2 can be found at http://www.geoscience.nt.gov.au/gemis/ntgsjspui/handle/1/79405.
