# Supplementary material for: Dynamic interaction between basin redox and the biogeochemical nitrogen cycle in an unconventional Proterozoic petroleum system
Source: Sci Rep. 2019 Mar 26;9:5200. doi: 10.1038/s41598-019-40783-4 (PMC6435709; doi:10.1038/s41598-019-40783-4)
Supplement: Supplementary file 1 — Supplementary Information [file 41598_2019_40783_MOESM1_ESM.docx]

Supplementary material for:

Dynamic interaction between basin redox and the biogeochemical nitrogen cycle in an unconventional Proterozoic petroleum system

Grant M. Cox^1*^, Pierre Sansjofre^2^, Morgan L. Blades^1^, Juraj Farkas^1^, Alan S. Collins^1^

* Corresponding author

1. Centre for Tectonics Resources and Exploration (TRaX), Department of Earth Sciences, University of Adelaide, Adelaide, S.A., 5005, Australia.

2. UMR 6538, Laboratoire Géosciences Océan, Institut Universitaire Européen de la Mer, rue Dumont d’Urville, 29280 Plouzané, France.

Corresponding author address: [grant.cox@adelaide.edu.au](mailto:grant.cox@adelaide.edu.au)

*Fast fourier transform (FFT) analysis*

We conducted time series analysis on the geochemical record preserved within the Altree 2 core using the FFT (Cooley and Tukey, 1965) in order to test for the presence of any significant cycles. For the FFT, the spectral power used is the complex conjugate of the Fourier coefficients, normalized to unit mean power (Muller and MacDonald, 2000). We evaluated the significance of the FFT spectral peaks using a Monte Carlo routine to simulate noise (Muller and MacDonald, 2000). FFTs were performed on each of these 1000 randomly generated time series; a 95% confidence level was typically approximated for each frequency by calculating three times the mean power (Muller and MacDonald, 2000). Spectral peaks rising above this 95% confidence level are statistically significant (Fig. 1). For 𝛿^15^N, 𝛿^13^C, TOC and Mo, peaks associated with cycles at a wavelength of ~110 rise above our estimation of noise making them statistically significant (Fig. 1).


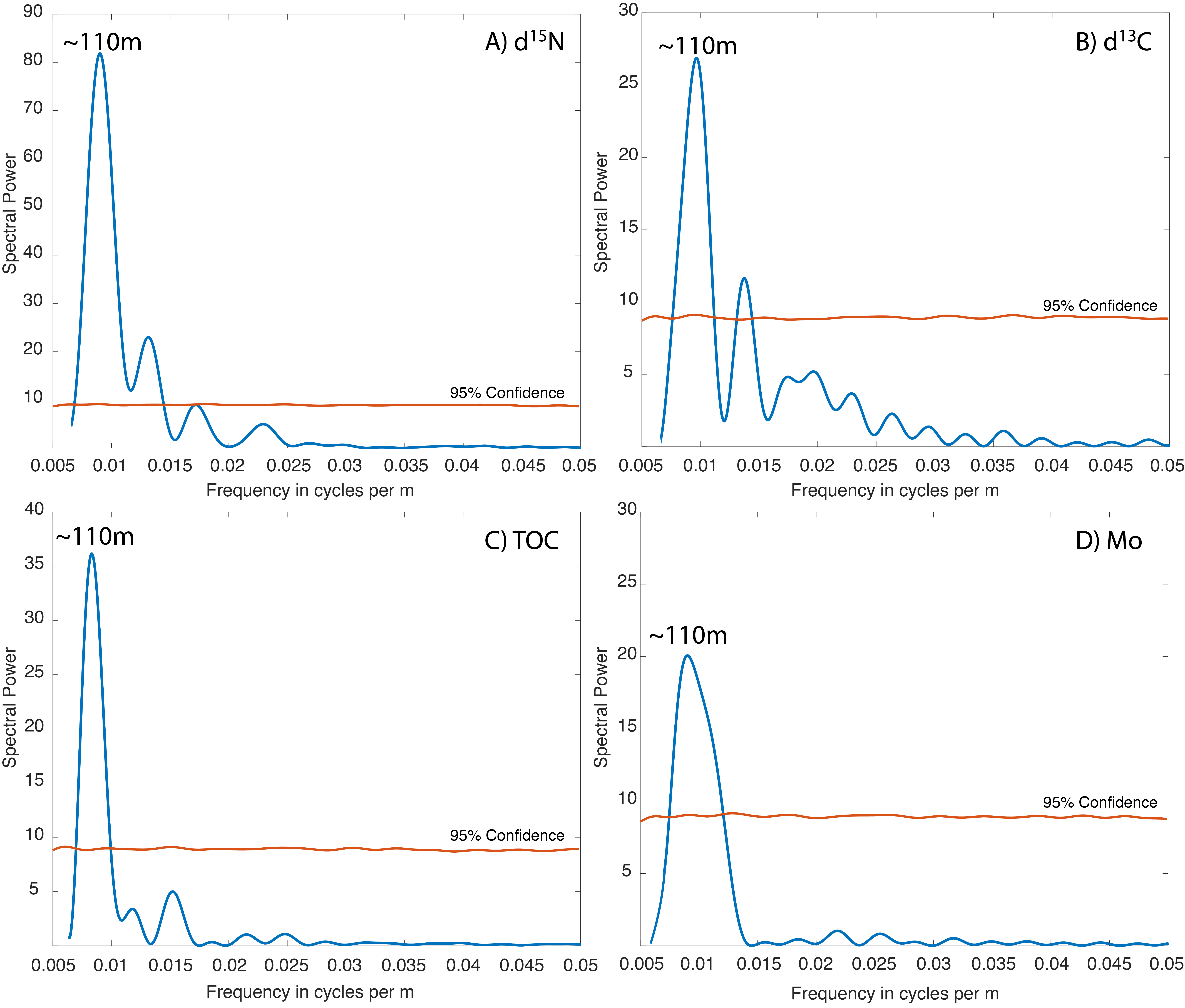


Figure 1. Fast-Fourier transform results for A) 𝛿^15^N, B) 𝛿^13^C, C) TOC and D) Mo. Peaks rising above noise occur consistently at a wavelength of ~ 110m making these cycles unlikely to be random features.

Table 1. Whole rock pyrolysis results.

Table 2. Whole rock XRD results.

Table 3. Nitrogen and carbon data plus elemental abundances for V, Mo, P_2_O_5_, TiO_2_, K_2_O and Al_2_O_3_. Major element and trace element data are reproduced from Ref

References

Cooley, J. W., and Tukey, J. W., 1965, An Algorithm for the Machine Calculation of Complex Fourier Series: Mathematics of Computation, v. 19, no. 90, p. 297-301.

Cox, G. M., Jarrett, A., Edwards, D., Crockford, P. W., Halverson, G. P., Collins, A. S., Poirier, A., and Li, Z.-X., 2016, Basin redox and primary productivity within the Mesoproterozoic Roper Seaway: Chemical Geology, v. 440, p. 101-114.

Hall, L. S., Boreham, C. J., Edwards, D. S., Palu, T. J., Buckler, T., Hill, A. J., and Troup, A., 2016, Cooper Basin Source Rock Geochemistry: Regional Hydrocarbon Prospectivity of the Cooper Basin, Part 2.: Record 2016/06. Geoscience Australia, Canberra.

Muller, R. A., and MacDonald, G. J., 2000, Ice ages and astronomical causes; data, spectral analysis and mechanisms, Chichester, United Kingdom, Praxis Publishing, 318 p.:
